# Pollen viabilities and gene expression profiles across *Musa* genomes

**DOI:** 10.1093/aobpla/plad052

**Published:** 2023-07-31

**Authors:** Yonlada Mingmanit, Thanita Boonsrangsom, Kawee Sujipuli, Kumrop Ratanasut, Phithak Inthima

**Affiliations:** Department of Agricultural Science, Faculty of Agriculture Natural Resources and Environment, Naresuan University, 99 Moo 9, Tha Pho, Phitsanulok 65000, Thailand; Department of Agricultural Science, Faculty of Agriculture Natural Resources and Environment, Naresuan University, 99 Moo 9, Tha Pho, Phitsanulok 65000, Thailand; Center of Excellence in Research for Agricultural Biotechnology, Naresuan University, 99 Moo 9, Tha Pho, Phitsanulok 65000, Thailand; Department of Agricultural Science, Faculty of Agriculture Natural Resources and Environment, Naresuan University, 99 Moo 9, Tha Pho, Phitsanulok 65000, Thailand; Center of Excellence in Research for Agricultural Biotechnology, Naresuan University, 99 Moo 9, Tha Pho, Phitsanulok 65000, Thailand; Department of Agricultural Science, Faculty of Agriculture Natural Resources and Environment, Naresuan University, 99 Moo 9, Tha Pho, Phitsanulok 65000, Thailand; Center of Excellence in Research for Agricultural Biotechnology, Naresuan University, 99 Moo 9, Tha Pho, Phitsanulok 65000, Thailand; Center of Excellence in Research for Agricultural Biotechnology, Naresuan University, 99 Moo 9, Tha Pho, Phitsanulok 65000, Thailand; Plant Tissue Culture Research Unit, Department of Biology, Faculty of Science, Naresuan University, 99 Moo 9, Tha Pho, Phitsanulok 65000, Thailand

**Keywords:** Aceto orcein, banana, pollen germination, RT–qPCR, TTC test

## Abstract

Banana (*Musa* spp.) is a major global economic fruit crop. However, cross-pollination from other *Musa* cultivars grown in nearby plantations results in seeded fruit that exceeds market demand. This study investigated pollen viability and germination and examined the expression profiles of pollen development-related genes across seven *Musa* genomes (AA, BB, AAA, BBB, AAB, ABB and ABBB). Twenty-three *Musa* cultivars were assessed for pollen viability using lacto-aceto-orcein and triphenyltetrazolium chloride staining methods. Results revealed that pollen viability obtained from both methods was significantly different among all the studied cultivars. Cultivars carrying BB (diploid) genomes had higher viability percentages than AA (diploid), AAA, BBB, AAB and ABB (triploid) and ABBB (tetraploid) genomes. Germination of the studied cultivars was also investigated on pollen culture medium, with results showing significant differences between the pollen of each cultivar. The best germinating cultivar was TKM (11.0 %), carrying BB genome. Expression profiles of pollen development-related genes by RT–qPCR indicated that both *TPD1A* and *MYB80* genes were highly expressed in triploid *Musa* genomes but the *PTC1* gene showed down-regulated expression, resulting in non-viable pollen. Pollen viability, pollen germination and pollen development-related genes differed across *Musa* cultivars. This knowledge will be useful for the selection of male parents for *Musa* cross-breeding programs. Pollen viability should also be considered when planning *Musa* production to avoid seeded fruit.

## Teaser

Bananas (*Musa* genus) possess high pollen viability and germination rates, resulting in the production of seeded fruit, which is undesirable for the market. Our study found that bananas with a BB genome revealing exceptionally elevated pollen viability and germination rates compared to other variants. Moreover, we investigated the expression of three pollen development-related genes. Notably, the TPD1A and MYB80 genes showed significantly high expression in bananas with a triploid genome. However, the PTC1 gene exhibited down-regulated expression, suggesting its role in promoting non-viable pollen. These findings hold significant promise for selecting superior male parents in cross-breeding programmes. By considering pollen viability, banana production can be optimized to meet market demands for seedless fruit and avoid the presence of unwanted seeded varieties.

## Introduction

Banana (*Musa* spp.), belonging to the monocotyledon Musaceae family, originated in Southeast Asia (Martanti *et al*. 2022) and is one of the important economic fruit crops in Thailand (Arwatchananukul *et al*. 2022). Most *Musa* cultivars have been improved by mutation, natural selection and artificial breeding (Jeensae *et al*. 2020) through intraspecific or interspecific hybridization between two wild-diploid *Musa* hybrids, *Musa acuminate* (carrying AA genome 2*n* = 22) and *Musa balbisiana* (carrying BB genome, 2*n* = 22). These hybrids have resulted in high genetic diversity among *Musa* cultivars. All *Musa* cultivars can be divided into three genomic groups based on polyploid level as diploid (AA, BB and AB), triploid (AAA, BBB, AAB and ABB) and tetraploid (AAAA, ABBB and AABB) (De Langhe *et al*. 2010). Cultivated edible *Musa* are seedless vegetative parthenocarpic fruits with pollination but not fertilization, whereby the ovary develops by pollinating non-viable pollen (Backiyarani *et al*. 2021). In Thailand, *Musa* ‘Kluai Khai’ (AA), ‘Kluai Hom’ (AAA) and ‘Kluai Namwa’ (ABB) are popularly grown for commercial production with high market demand because seedless fruit has a good taste and sweet smell and is viable for industrial processing. However, *Musa* plantations often face problems with seed formation caused by cross-pollination from other *Musa* cultivars grown in nearby areas. The seeded *Musa* fruit has low market demand and is difficult to process. To solve this limitation, *Musa* pollen viability and development require study as a useful guideline to prevent seed formation and cross-breeding in mass *Musa* production to create the desired cultivars.

Seed formation in plants generally occurs through fertilization between female (egg) and male (pollen) parents, with success depending on two main mechanisms involved in pollen development (pollen viability) and pollen tube germination. *Musa* varieties contain A and B genomes and (diploid, triploid and tetraploid) polyploid levels that impact pollen viability and germination percentages. The diploid *Musa* genome has the highest pollen viability at 11 and 3 folds higher than triploid and tetraploid genomes, respectively (Fortescue and Turner 2004; Jeensae *et al*. 2020), while the diploid wild *Musa* (AA and BB genomes) has higher viability and pollen germination than triploid genomes (Adeleke *et al*. 2004; Soares *et al*. 2008; Oselebe *et al*. 2014). Reduced pollen viability results from abnormal processes during meiotic cell division, such as non-reducing chromosome segregation of trivalent or tetravalent pairings in anaphase I (Šimoníková *et al*. 2020).

The staining of pollen using triphenyltetrazolium chloride (TTC) and lacto-aceto-orcein (LAO) has been widely employed to assess pollen viability due to its simplicity and effectiveness (Munhoz *et al*. 2008; Coelho *et al*. 2012; Damaiyani and Hapsari, 2018). Triphenyltetrazolium chloride starts off colourless but turns red when metabolically active pollen undergoes reduction through dehydrogenase enzymes, indicating viability. In contrast, LAO stains chromosomes, exhibiting a red colour that provides insights into their viability. However, there is limited research comparing these two methods across different *Musa* genomes.

Few reports have assessed the molecular mechanisms of pollen development and formation in *Musa*. Hu *et al*. (2020) found that pollen development in *Musa itinerans* (*Ma*) was significantly regulated by the functions of three key genes: *tapetum determinant 1* (*MaTPD1A*), *persistent tapetal cell 1* (*MaPTC1*) and *myeloblastosis 80* (*MaMYB80*). The expression of the *MaTPD1A* gene plays an important role in pollen development by controlling the expression of *MaMYB80* and *MaPTC1* genes in male flowers. The up-regulated *MaTPD1A* gene promotes high expression of the *MaMYB80* gene but reduces the expression of the *MaPTC1* gene, resulting in pollen sterility in male flowers, producing small seedless fruit. However, the expression profile of the *MaTPD1A* gene in mediating pollen development has not been studied in *Musa* cultivars of Thai germplasm.

This research determined pollen viability by TTC and LAO staining and *in vitro* germination, and expression profiles of pollen development-related genes across *Musa* genome groups (AA, BB, AAA, BBB, AAB, ABB and ABBB). Increased knowledge of pollen viability can benefit the selection of male parents for *Musa* cross-breeding programs to produce seedless fruit.

## Materials and Methods

### Plant materials

Twenty-three Thai *Musa* cultivars consisting of seven genomes (3 AA, 3 BB, 4 AAA, 1 BBB, 4 AAB, 7 ABB and 1 ABBB) (Table 1) were used in this study. All cultivars were planted and conserved in germplasm at the Phitsanulok Agricultural Extension and Development Center, Phitsanulok Province, Thailand. At 30 days post-flowering, the male inflorescence sample of each cultivar was collected during the anthesis stage in the morning between 0800 and 0900 h, and the sample was stored on ice until used, following the procedure of Soares *et al*. (2015).

**Table 1.** Thai *Musa* cultivars used in this study.

No.	Botanical name ‘Cultivar’	Code	Genome
**1**	*Musa* ‘Kluai Leb Mu Nang’	LMN	AA
**2**	*Musa* ‘Kluai Nam Thai’	NT	AA
**3**	*Musa* ‘Kluai Khai Kamphaengphet’	KKP	AA
**4**	*Musa* ‘Tani Buri Ram167’	T167	BB
**5**	*Musa* ‘Tani A15’	TA15	BB
**6**	*Musa* ‘Tani Kip Ma’	TKM	BB
**7**	*Musa* ‘Kluai Khai Pratabong’	KPB	AAA
**8**	*Musa* ‘Kluai Nak’	NAG	AAA
**9**	*Musa* ‘Kluai Hom Khieo’	HK	AAA
**10**	*Musa* ‘Kluai Hom Thong’	HT	AAA
**11**	*Musa* ‘Kluai Lep Chang Kut’	LCK	BBB
**12**	*Musa* ‘Kluai Roiwi’	RV	AAB
**13**	*Musa* ‘Kluai Nom Sao’	NSA	AAB
**14**	*Musa* ‘Kluai Nom Sawan’	NSW	AAB
**15**	*Musa* ‘Kluai Niu Jorakhe’	NCD	AAB
**16**	*Musa* ‘Thepanom’	TPN	ABB
**17**	*Musa* ‘Hak Muk’	HM	ABB
**18**	*Musa* ‘Kluai Namwa Nuan Chan’	NNJ	ABB
**19**	*Musa* ‘Pakchong 50’	NPC50	ABB
**20**	*Musa* ‘Kluai Namwa Mali Ong’	NMO	ABB
**21**	*Musa* ‘Kluai Namwa Kap Khao’	NKK	ABB
**22**	*Musa* ‘Kluai Namwa Dam’	ND	ABB
**23**	*Musa* ‘Theparod’	TPR	ABBB

### Assessment of *Musa* pollen viability by histochemical assay

**Figure 1.**
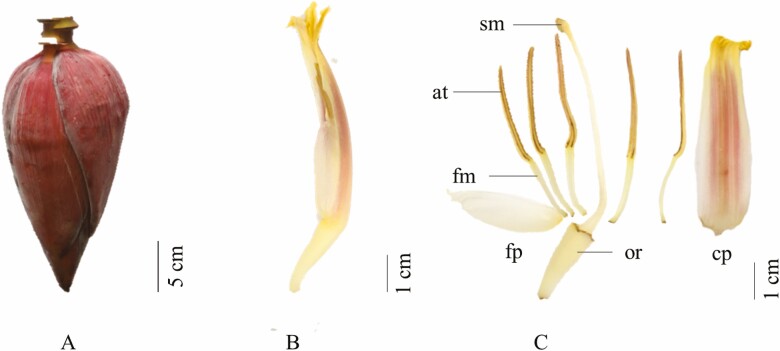
Morphology of young *Musa* inflorescence (30 days post-flowering) as one representative of cultivar NKK (listed in Table 1). Bracts and flowers of closed inflorescence (A), separated male flower (B), components of flowers (sm = stigma, at = anthers, fm = filament, fp = free tepal, or = ovary and cp = compound tepal) (C).

The histochemical analysis of *Musa* pollen viability was assessed by staining with two assays. The 1 % LAO assay acted as positively stained chromosomes, modified from Syakhril *et al*. (2019), while the 1 % 2,3,5-triphenyltetrazolium chloride assay acted as an enzymatic test by detecting the dehydrogenase activity in *Musa* pollen grains, modified from Damaiyani and Hapsari (2018). Healthy male flowers of *Musa* (Fig. 1B) were removed from the young inflorescence (Fig. 1A), with the anther compartment collected from the male flower (Fig. 1C) by forceps. For the LAO staining assay, individual whole anthers were transferred into 1.5 mL Eppendorf tubes containing 20 µL of 1 % LAO solution diluted in 45 % acetic acid. The anther wall was cut by a sterilized needle to release the pollen, which was immersed in LAO solution. The mixture was adjusted to a final volume of 1 mL with sterilized distilled water (980 µL) and incubated at room temperature for 5–10 min. The TTC histochemical assay procedure was similar to the LAO assay, except that 1 mL of 1 % TTC solution (in Tris buffer containing HCl 0.15 M, pH 7.8) was used instead of LAO solution, and the mixture was incubated at room temperature in the dark for 2 h.

To investigate pollen viability, a stained pollen mixture (100 µL) from individual assays was dropped on a glass slide and covered with a glass slip. Pollen viability was observed under a polarized light microscope (Olympus BX53M, Japan) with ×4 magnification and photographed at 25 fields per sample.

Images were recorded to count the total number of pollen grains and viable pollen grains. Pollen grains stained with either LAO or TTC that expressed as a round shape and were dark/light red were considered as viable, while pollen grains that expressed as an irregular or wrinkled shape that were pale red or transparent were considered non-viable.

Numbers of pollen grains from individual samples were collected from five anthers per *Musa* cultivar, while individual cultivars were performed for at least six biological replicates.

### Assessment of *Musa* pollen germination by *in vitro* assay

The assessment of *Musa* pollen germination was accomplished on pollen culture medium following the modified assay of Brewbaker and Kwack (1963). The medium consisting of 0.03 % calcium nitrate, 0.02 % magnesium sulfate, 0.01 % potassium nitrate, 0.01 % boric acid and 15 % sucrose with pH adjusted to 7.0 was sterilized by autoclaving at 121 °C for 15 min. Whole anthers of individual *Musa* samples were placed into 1.5 mL Eppendorf tubes containing 1 mL of sterilized pollen culture medium, and the pollen was isolated from its anther by cutting with a needle. The mixture was incubated at room temperature in the dark for 2 h. At the end of the culture period, the pollen was well mixed and 100 µL was dropped onto a glass slide and covered with a glass slip. Germination was observed under a polarized microscope (Olympus BX53M) with ×4 magnification and photographed at 25 fields per sample.

Images were taken to count the total number of pollen grains and number of germinated pollen grains (germinated pollen must have pollen tube length greater than or equal to pollen diameter). Pollen grains from the individual samples were collected from five anthers per *Musa* cultivar, and individual cultivars were performed for at least six biological replicates.

### Determining expression profiles of pollen development-related genes in *Musa*

Of the 23 studied *Musa* cultivars, 6 with high (T167 and TA15), medium (KKP and KPB) and low (NMO and NNJ) percentages of pollen viability were selected to determine the gene expression profiles involved in *Musa* pollen development.

### Total RNA extraction

Total RNA was extracted from the anther samples collected from six *Musa* cultivars, following the modified CTAB method of Khairul‑Anuar *et al*. (2019). Briefly, *Musa* anthers (100 mg) per sample were ground to fine powder with liquid nitrogen in a mortar and pestle. The powder was transferred into a 1.5 mL Eppendorf tube containing 800 µL of cetyltrimethylammonium bromide (CTAB) buffer (10 % CTAB, 1 M Tris–HCl pH 8.0, 5 M NaCl, 0.5 M EDTA pH 8.0 and 1 % PVP-6000). The mixture was gently vortexed, incubated at 65 °C for 45 min, allowed to cool at room temperature for 5 min and then centrifuged at 7000×g at 4 °C for 5 min. The supernatant was transferred into a new Eppendorf tube and an equal volume of phenol:chloroform:isoamyl alcohol (P:C:I in the ratio 25:24:1, pH 4.5) was added. The mixture was vortexed for 5 min, then centrifuged at 17 500×g at 4 °C for 15 min and the supernatant (top layer) was transferred to a new Eppendorf tube. An equal volume of C:I at a ratio of 24:1 was added, mixed gently by turning the tube back and forth several times, and then centrifuged at 17 500×g at 4 °C for 15 min. This step was repeated once by adding an equal volume of C:I ratio (24:1). The supernatant (top layer) was then transferred to a new Eppendorf tube and 10 M LiCl at 1/3 volume was added to the supernatant. The mixture was mixed well by inverting the tube and incubated at 4 °C overnight. It was then centrifuged at 17 500×g at 4 °C for 30 min. The suspension was discarded and the total RNA pellet was washed and collected two times with 500 µL of 70 % ethanol by centrifuging at 17 500 × g at 4 °C for 15 min. The total RNA pellet was air-dried for 10 min and dissolved in 30 μL of RNase-free water. Subsequently, total RNA was assessed for quality and integrity by gel electrophoresis, with concentration quantified by absorbance measurements at A260 and A280 nm using a NanoDrop Lite Spectrophotometer (Thermo Fisher Scientific, Waltham, MA, USA). Purity, assessed by the ratio of A260 nm and A280 nm, ranged from 1.8 to 2.0. The total RNA was treated with DNase l (Thermo Fisher Scientific, Waltham, MA, USA) to remove genomic DNA contamination according to the manufacturer’s instructions.

### First-strand cDNA synthesis

The total RNA template (500 ng) was reversed to the first strand cDNA in a 20 µL reaction mixture using a RevertAid First Strand cDNA Synthesis Kit (Thermo Fisher Scientific, Baltics, UAB, Lithuania) containing 1 µL Oligo (dT)_18_ primer, 4 µL 5X Reaction buffer, 1 µL RiboLock RNase Inhibitor (20 U µL^−1^), 2 µL 10 mM dNTP Mix and 1 µL RevertAid M-MuLV RT (200 U µL^−1^), with the final volume adjusted to 20 µL using nuclease-free water. The reaction mixture was incubated as three steps of 25 °C for 5 min, 42 °C for 60 min and 70 °C for 5 min. The first strand cDNA was immediately subjected to RT–qPCR amplification and stored at −80 °C until required for further use.

### RT–qPCR analysis

Four primer pairs designed by the Oligo Analyzer program (version 1.2, USA) (Table 2) were used to evaluate the expression profiles of the three candidate genes associated with pollen development including *TPD1A* (*tapetum determinant 1*), *PTC1* (*persistent tapetal cell 1*) and *MYB80* (*myeloblastosis 80*), while the *CAC* (*Clathrin adaptor complexes medium*) gene was used as the internal control reference for normalization.

**Table 2.** Primer sequences used in this study.

GenBank accession number	Primer name	Primer sequences (5ʹ-3ʹ)	Amplicon size (bp)
HQ853240	*CAC*-F	CTCCTATGTTGCTCGCTTATG	146
	*CAC*-R	GGCTACTACTTCGGTTCTTTC	
XM_009388561.1	*TPD1A*-F	GACAGCTCAATCGAGGAAG	113
	*TPD1A*-R	CCTCTCCGGTCATCTCCATC	
XM_009416075.2	*PTC1*-F	AATCAGGAAGCAGCATCGTG	118
	*PTC1*-R	TCCTCCTTTCCACCACACA	
XM_009403755.1	*MYB80-*F	AAGCCGTTCTCCCATCTCAT	172
	*MYB80*-R	TGTAAGCGTTGCCTGTGAC	

The RT–qPCR reaction (12.5 µL) was performed in Thermo Scientific Maxima SYBR Green qPCR Master Mix (2X) (no ROX, Thermo Fisher Scientific, Baltics, UAB, Lithuania) (6.5 µL), 10 µM of each forward (0.5 µL) and reverse (0.5 µL) primer, first strand cDNA (1 µL) and nuclease-free water (4 µL), using the Eco48 Real-Time PCR system (Eco 48, PCRmax Limited, UK).

The RT–qPCR amplification was carried out under the following conditions: 1 cycle of 50 °C for 2 min and 1 cycle of 95 °C for 10 min, followed by 40 cycles of 95 °C for 15 s, 60 °C for 30 s and 72 °C for 30 s. Finally, a melting curve was realized by progressively heating the reaction mixture from 55 to 95 °C using 0.3 °C increments every 0.75 s to check the purity of the RT–qPCR product. All reactions were technically repeated five times with three biological replicates.

The baseline correction was automatically calculated to determine the cycle threshold (Ct) value in each reaction using Eco 48 Study Software installed in the instrument. Data were normalized with *CAC* as the endogenous control (set to 1). Relative expression of the three target genes was analysed using the comparative Ct method (ΔCt1, ΔCt2 and 2^−ΔΔCt^), where ΔCt1 represented different expression between target and reference gene in samples of five *Musa* cultivars (T167, TA15, KKP, KPB and NMO), ΔCt2 represented different expression between target and reference genes in NNJ samples and ΔΔCt showed different expression levels between ΔCt1 and ΔCt2. Normalized target gene expression level was calculated by the comparative Ct method (2^−ΔΔCt^) using Microsoft Excel 2010 program.

### Data analysis

Pollen viability percentage was calculated using the formula of Ssebuliba *et al*. (2008).


$$\begin{array}{l} \text{Pollen viability }(\%)\\ =\frac{\text{number of stained pollen grains}}{\text{total number of pollen grains }}\times 100 \end{array}$$

Pollen germination percentage was calculated by the formula of Nyine and Pillay (2007).


$$\begin{array}{l} \text{Pollen germination (}\%)\;=\\ \frac{\text{number of germinated pollen grains}}{\text{total number of pollen grains}}\times 100 \end{array}$$

The diameters in micrometres of viable and non-viable pollen grains were measured using the ImageJ program (version 1.52, USA).

Pollen tube length in micrometres was measured using the ImageJ program (version 1.52, USA).

All data (pollen viability, pollen germination and gene expression) were statistically assessed using ANOVA. Mean comparisons between multiple treatments (*Musa* genotypes) were assessed by Duncan’s Multiple Range Test (DMRT) at *P*-value ≤ 0.1 statistical significance using the Statistical Product and Service Solution version 17.0 software (SPSS Inc.; 2008, Chicago, USA). All values were expressed as mean ± standard error (SE) of three to six biological replicates.

## Results

### Assessment of *Musa* pollen viability by histochemical assay

**Figure 2.**
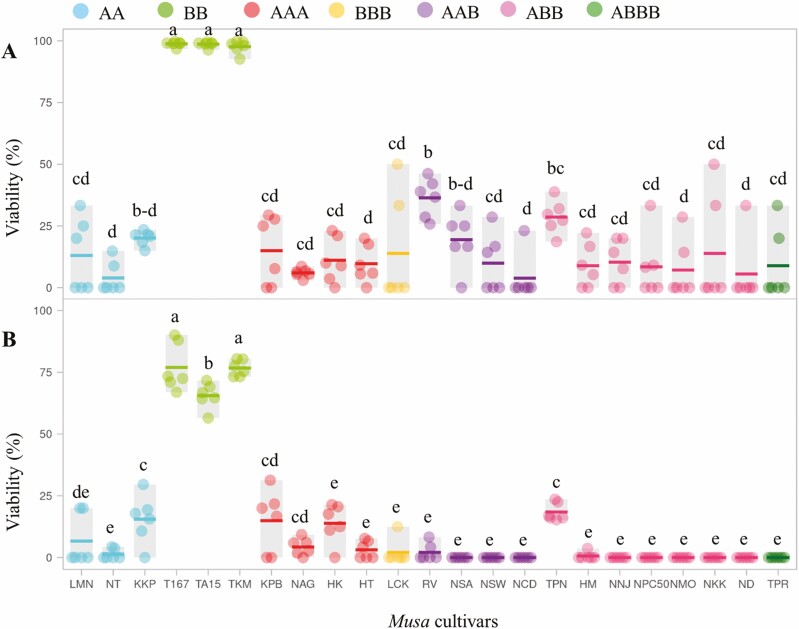
Pollen viability percentage among 23 *Musa* cultivars stained with either LAO (A) or TTC (B) assay. The dot represents individual data, horizontal line displays mean (*n* = 6 flowers) and grey box indicates the range, different letters in individual box indicate significant differences analysed by Duncan’s Multiple Range Test at *P* ≤ 0.01.

Pollen viability among the 23 *Musa* cultivars (listed in Table 1) was quantified by staining with 1 % LAO assay and 1 % TTC assay. Both assays gave similar results of pollen viability for all the studied *Musa* cultivars. The LAO staining assay provided higher sensitivity for pollen viability in most *Musa* cultivars, except for the HK cultivar compared to the TTC assay (Fig. 2A and B).

**Figure 3.**
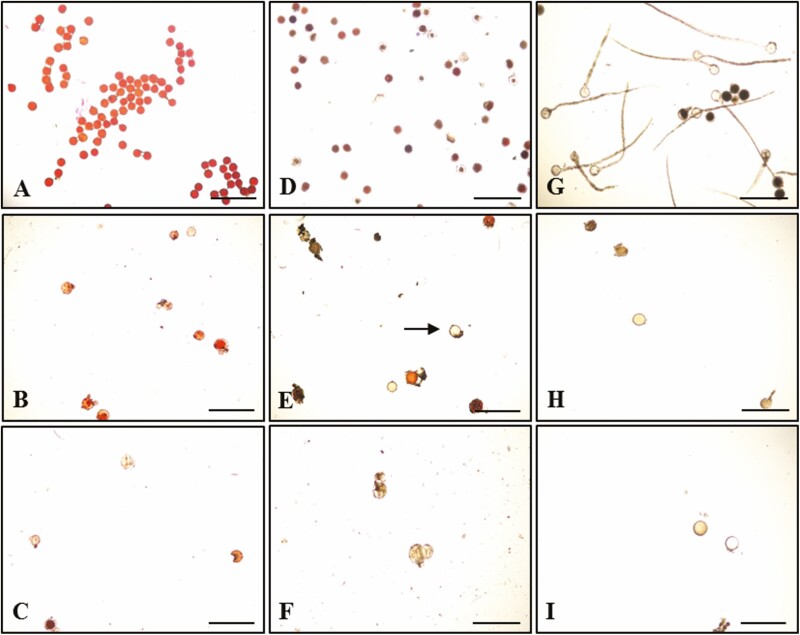
Viability and germination percentage of *Musa* pollen. High percentage viability level (T167) (A and D), medium (KKP) (B and E) and low (HM) (C and F), after staining with LAO and TTC, respectively. Viable pollen shows as red with non-viable pollen clear. High percentage of pollen germination (TKM) (G), low (KKP) (H) and non-germination (KPB) (I). Germinated pollen must have pollen tube length greater than or equal to pollen diameter. Bars indicate 500 µm.

For the LAO staining assay, pollen viability percentages of all studied *Musa* cultivars were statistically significantly different, ranging from 3.8 ± 3.8 to 98.7 ± 0.4 (Fig. 2A). The cultivars were divided into three groups according to modified criteria from Damaiyani and Hapsari (2018) as high (51–100 %), medium (16–50 %) and low (0–15 %) pollen viability. Three cultivars (T167, TA15 and TKM) belonging to the BB genome gave the highest levels of pollen viability as 98.7, 98.6 and 97.6 %, respectively (Figs. 2A and 3A), while four cultivars (RV, TPN, KKP and NSA), belonging to AAB, ABB and AA genomes had moderate percentages of pollen viability at 36.4, 28.6, 20.1 and 19.4 %, respectively (Figs. 2A and 3B). The remaining (16) *Musa* cultivars had low levels of pollen viability percentage, ranging from 3.8 to 14.9 % (Figs. 2A and 3C). Sixteen *Musa* cultivars exhibited low pollen viability, while nine (TPR, ND, NKK, NMO, NPC50, NNJ, NCD, NSW and NSA) showed complete male sterility with non-pollen viability detected by TTC assay (Figs. 2B and 3F).

**Figure 4.**
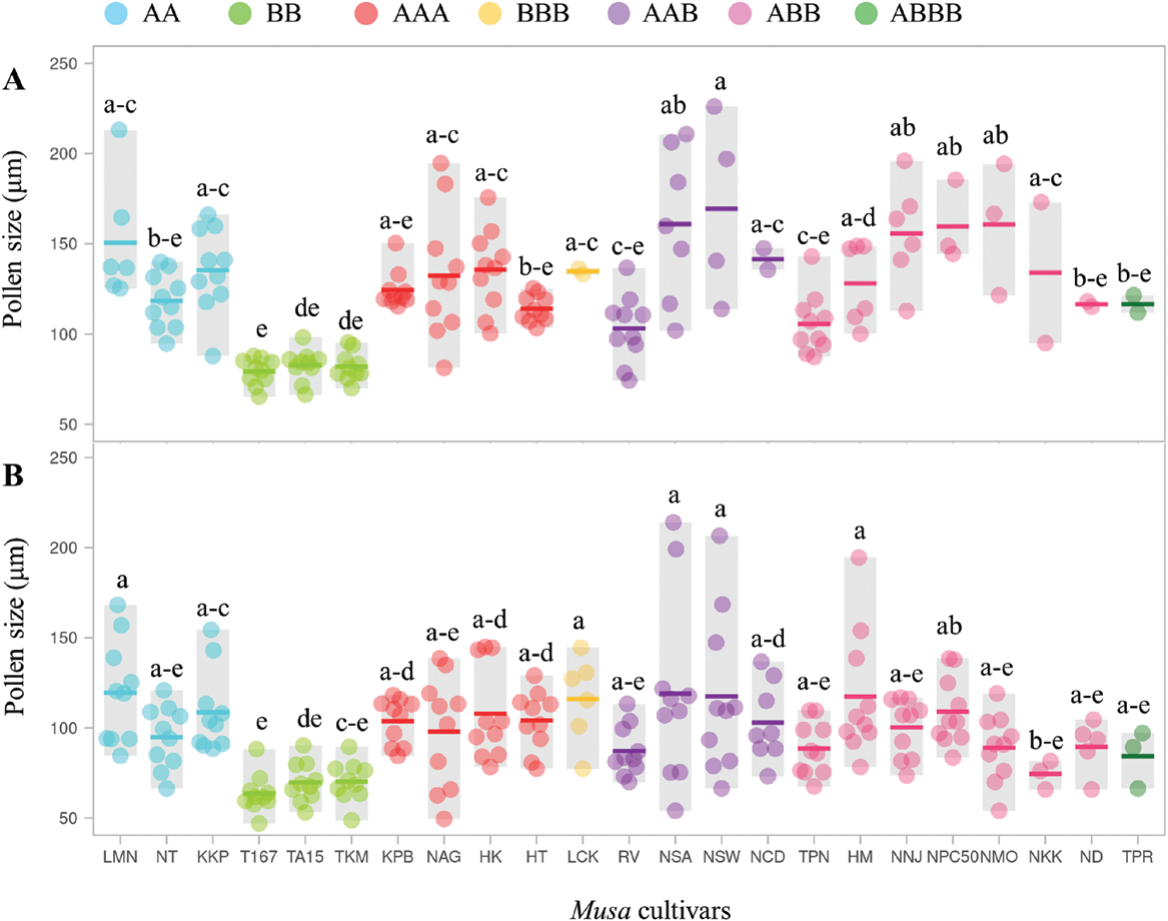
Viable (A) and non-viable (B) pollen sizes among the studied 23 *Musa* cultivars. The dot represents individual data, horizontal line displays mean (*n* = 10 pollen grains) and grey box indicates the range, different letters in individual box indicate significant differences analysed by Duncan’s Multiple Range Test at *P* ≤ 0.01.

When comparing the size of the pollen grains (µm) under a polarized light microscope, viable pollen grain diameter (µm) in all studied *Musa* cultivars was significantly larger than non-viable pollen. Most *Musa* cultivars had pollen grains larger than 100 µm, except for three (T167, TA15 and TKM) with pollen grains smaller than 100 µm (Fig. 4A and B).

Pollen size was related to the pollen viability percentage. The *Musa* cultivars carrying the BB genome (T167, TA15 and TKM) showed highest percentages of pollen viability (98.3 % in LAO and 73.1 % in TTC assay) and number of pollen grains per flower (11 682). By contrast, the *Musa* cultivar TPR, belonging to ABBB genome had the lowest percentage of pollen viability (8.9 % in LAO and 0 % in TTC assay), with pollen grains per flower (16.1) (Table 3).

**Table 3.** Pollen viability and germination among the 23 tested Thai *Musa* cultivars. Data are mean ± SE of six biological replicates. Different letters within the same column indicate significant differences analysed by Duncan’s Multiple Range Test at *P* ≤ 0.01. N/A (not applicable) indicates pollen non-germination.

*Musa* cultivar	Genomic group	Pollen grains per flower	Germination (%)	Pollen tube length (µm)
LMN	AA	35.0 ± 5.9 e	0.0 ± 0.0 b	N/A
NT	AA	312.2 ± 48.1 e	0.9 ± 0.9 b	137.2 ± 137.2 d
KKP	AA	575.6 ± 44.1 e	3.4 ± 1.2 b	496.6 ± 208.0 b
T167	BB	9407.2 ± 269.3 c	2.1 ± 0.6 b	802.4 ± 802.4 a
TA15	BB	14 953.9 ± 665.1 a	2.1 ± 1.0 b	413.8 ± 117.8 bc
TKM	BB	10 685.0 ± 437.1 b	11.0 ± 1.4 a	645.5 ± 27.2 ab
KPB	AAA	217.2 ± 37.5 e	0.0 ± 0.0 b	N/A
HK	AAA	196.1 ± 24.3 e	0.0 ± 0.0 b	N/A
HT	AAA	320.6 ± 68.7 e	0.0 ± 0.0 b	N/A
NAG	AAA	612.2 ± 51.4 e	1.0 ± 0.7 b	148.6 ± 94.8 d
LCK	BBB	28.9 ± 1.5 e	0.0 ± 0.0 b	N/A
RV	AAB	208.3 ± 12.3 e	0.0 ± 0.0 b	N/A
NSA	AAB	35.6 ± 5.1 e	0.0 ± 0.0 b	N/A
NSW	AAB	34.4 ± 7.4 e	0.0 ± 0.0 b	N/A
NCD	AAB	6.1 ± 1.8 e	0.0 ± 0.0 b	N/A
TPN	ABB	2320.6 ± 254.9 d	0.8 ± 0.3 b	208.2 ± 71.7 cd
HM	ABB	191.1 ± 42.2 e	0.0 ± 0.0 b	N/A
NNJ	ABB	66.1 ± 8.0 e	0.0 ± 0.0 b	N/A
NPC50	ABB	63.9 ± 5.1 e	0.0 ± 0.0 b	N/A
NMO	ABB	38.3 ± 6.5 e	0.0 ± 0.0 b	N/A
NKK	ABB	12.2 ± 2.5 e	0.0 ± 0.0 b	N/A
ND	ABB	23.3 ± 3.8 e	0.0 ± 0.0 b	N/A
TPR	ABBB	16.1 ± 5.9 e	4.2 ± 4.2 b	104.0 ± 104.0 d

### Assessment of *Musa* pollen germination by *in vitro* assay

*Musa* pollen germination was investigated on pollen culture medium following the modified assay of Brewbaker and Kwack (1963). Results showed that each *Musa* cultivar had significantly different pollen germination percentages. *Musa* cultivar TKM gave the highest germination percentage at 11.0 % (Fig. 3G), while seven *Musa* cultivars (TPR, KKP, TA15, T167, NAG, NT and TPN) showed low pollen germination percentage (Fig. 3H) as 4.2, 3.4, 2.1, 2.1, 1.0, 0.9 and 0.8 %, respectively. Fifteen *Musa* cultivars including LMN, KPB, HK, HT, LCK, RV, NSA, NSW, NCD, HM, NNJ, NPC50, NMO, NKK and ND did not completely germinate on the culture medium (Fig. 3I).

Among the germinated *Musa* cultivars, T167 had the longest average pollen tube length at 802.4 µm (Table 3), while TKM, KKP, TA15, TPN, NAG, NT and TPR gave average length of pollen tube as 645.5, 496.6, 413.8, 208.2, 148.6, 137.2 and 104.0 µm, respectively (Table 3).

### Assessment of gene expression involved in *Musa* pollen development

Previous results indicated that pollen viability percentages across the studied *Musa* cultivars by LAO staining assay could be classified into three groups.

**Figure 5.**
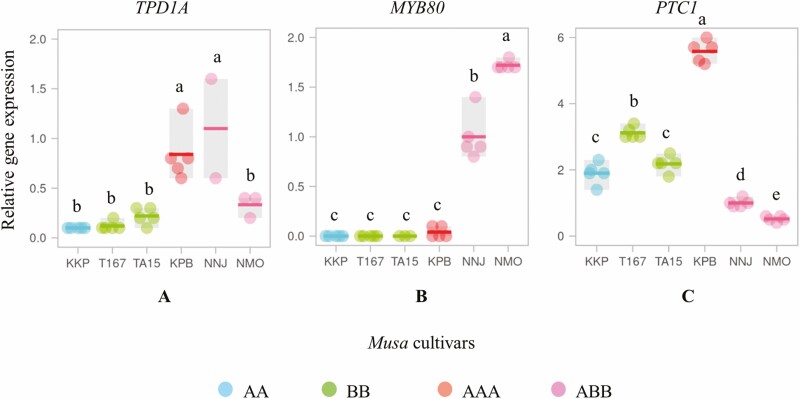
Expression of *TPD1A* (A), *MYB80* (B) and *PTC1*(C) genes in six *Musa* cultivars. The dot represents individual data, horizontal line displays mean (*n* = 5 technical iterations) and grey box indicates the range, different letters in individual box indicate significant differences analysed by Duncan’s Multiple Range Test at *P* ≤ 0.01. Data were normalized with the *CAC* reference gene as the endogenous control (set to 1).

Pollen viabilities were regulated by genes involved in the pollen development process. To test this hypothesis, three levels of pollen viability percentage across six *Musa* cultivars derived from high (T167 and TA15, carrying BB genome), medium (KKP and KPB, carrying AA and AAA genome, respectively) and low (NMO and NNJ, carrying genome ABB) were selected to analyse the expression profiles of *TPD1A*, *MYB80* and *PTC1* genes by RT–qPCR assay. All data obtained from these target genes were normalized by the *CAC* reference gene. Overall, the *TPD1A* gene (initial pathway of pollen development in microspore mother cell stage) in two *Musa* cultivars (NNJ and KPB, carrying ABB and AAA genome, respectively) showed higher expression, with statistically significant differences from the other four *Musa* cultivars (NMO, ABB genome; T167, TA15, BB genome; and KKP, AA genome) (Fig. 5A).

For genes associated with the middle pathway of pollen development (microspore stage), the *MYB80* gene showed the highest up-regulation in *Musa* cultivar NMO (ABB genome), followed by *Musa* cultivar NNJ (AA genome), which was significantly different at *P*-value ≤ 0.01 (Fig. 5B). The *MYB80* gene had highly decreased expression with statistically significant differences among the four *Musa* cultivars (T167, TA15, BB genome; KKP, AA genome; and KPB, AAA genome) (Fig. 5B).

The *PTC1* gene expression, required in the late stage of pollen development (mature pollen stage), was highly up-regulated in all cultivars with statistical differences. Among the six *Musa* cultivars, KPB with AAA genome had the highest *PTC1* gene expression followed by *Musa* cultivar T167, which was significantly different from the TA15 cultivar from the BB genome and KKP from the AA genome (Fig. 5C). Two *Musa* cultivars (NNJ and NMO, genome ABB) gave the lowest *PTC1* gene expression (Fig. 5C).

In summary, the *TPD1A* and *MYB80* gene expressions in *Musa* triploid genome (ABB and AAA) strongly increased compared to the diploid genome (AA and BB) (Fig. 5A and B). By contrast, the *PTC1* gene expression in the *Musa* triploid genome was lower than the diploid genome (Fig. 5C). The relationship of these genes suggested that high expression of either the *TPD1A* or *MYB80* gene might decrease the expression of *PTC1* gene, resulting in male sterility (non-viable pollen development). Conversely, low *TPD1A* or *MYB80* expression might not decrease expression of the *PTC1* gene, suggesting pollen grain development as male fertility.

## Discussion

*Musa* inflorescences contain two types of female and male flowers. Of these, the male-fertile *Musa* with viable pollen enabled cross-pollination with the female ovary, resulting in fully seeded fruit that was surplus to commercial market demands but a valuable source for further *Musa* cultivar improvement in cross-breeding programs (Ortiz and Swennen 2014). In recent years, pollen development and pollen vitality (Damaiyani and Hapsari 2018), including pollen tube germination (Waniale *et al*. 2021) in *Musa* have been reported as highly diverse across different genomes. Most pollen developments were studied in *Musa* AA and AAA genomes but not in the remaining *Musa* genomes. This research investigated pollen viability among seven *Musa* genomes. Pollen viability, obtained from LAO and TTC staining assay across 23 *Musa* cultivars, showed similar percentages; however, the LAO staining assay gave higher percentage values than the TTC assay. One possible explanation was that the LAO (reddish dye) stained specific DNA molecules that were present throughout whole cells, such as mitochondrial and chloroplast DNAs in cytoplasm and chromosomes in the nucleus (Coelho *et al*. 2012; Robles and Quesada 2021). Most areas inside the pollen were stained as red all over the cytoplasm and nucleus after LAO was absorbed into the cell, while the TTC assay reflected only survival pollen because TTC reduction activity is based on the dehydrogenase function in only living plant cells. Hydrogen ions released from respiration in survival cells reduced colourless TTC that turned into 2,3,5-triphenyl-tetrazolium-formazan with red appearance (Damaiyani and Hapsari 2018). These results concurred with previous findings that the LAO staining test gave a higher percentage of pollen viability than TTC staining, such as in *Nepenthes* spp. (Kaewkhumpai 2019) and *Crotalaria juncea* L. (Coelho *et al*. 2012). Thus, the TTC staining assay was more effective and suitable for evaluating pollen survival viability of *Musa* samples. These findings were supported by observations that pollen viability values determined by the TTC staining assay gave consistent results of pollen germination in banana (Soares *et al*. 2016) and *Solanum erianthum* (Ghosh *et al*. 2020). Therefore, in this experiment, we considered the results of TTC as an indication of viability.

As reported previously, pollen viability levels among different *Musa* genotypes can be divided into three groups of high (51–100 %), medium (16–50 %) and low (0–15 %) (Damaiyani and Hapsari 2018). From TTC, three *Musa* cultivars (T167, TA15 and TKM) belonging to the BB genome gave the highest levels of pollen viability at 77.0, 65.5 and 76.7 %, respectively, while two cultivars (TPN and KKP) belonging to ABB and AA genomes had moderate percentages of pollen viability at 18.4 and 16.0 %, respectively. The remaining 18 cultivars exhibited low pollen viability. Results indicated that *Musa* cultivars carrying diploid genomes (AA and BB) had higher pollen viability than *Musa* cultivars containing triploid genomes. Similarly, the pollen viability of diploid *M. acuminata* ‘Pisang Rejang’ (AA) was higher than triploid, tetraploid and mixoploids (Martanti *et al*. 2022). Diploid *Musa* enabled normal meiotic chromosome behaviour with bivalent chromosome pairs and normal balanced genomic segregation, resulting in fertile pollen (Damaiyani and Hapsari 2018; Ahmad *et al*. 2021). By contrast, high polyploidy with different genome (A and B) compositions in *Musa* might produce abnormal pollen due to partial homoeologous chromosome pairing of A and B during the prophase I of meiotic cell division (Jeridi *et al*. 2011; Ngatat *et al*. 2022), including non-reducing chromosome segregates of trivalent or tetravalent pairings in anaphase I, leading to unbalanced genome transmission in gametic cells (Šimoníková *et al*. 2020).

Interestingly, our results showed that LMN, NT and KKP cultivars with AA diploid genomes had different levels of pollen viability. These cultivars were produced by interbreeding of *Musa acuminate* parents with different diploid genomic groups (such as A1 and A2 chromosomes) (Jeensae *et al*. 2020). The *Musa* BB diploid had a higher percentage of viable pollen than the AA diploid, indicating that the genomic composition (A and/or B genome) of *Musa* impacted production of either partially sterile or fertile pollen. This insight into male sterility located on *Musa* A and/or B genome requires further investigation.

Pollen size across the 23 *Musa* cultivars was not related to pollen viability, concurring with previous findings that pollen size (most non-viable pollen) in triploid and tetraploid *Musa* was larger than diploid *Musa* (most viable pollen) (Panda *et al*. 2019; Sukkaewmanee 2019; Martanti *et al*. 2022). One reason given for this was that pollen size was affected by amounts of nuclear DNA contents (C) with equal size of A and B genomes as various polyploidies across *Musa* genotypes. Previous publications suggested that nuclear DNA content could be used as an indicator of genomic constitution (De Jesus *et al*. 2013), supporting that higher DNA composition positively related to longer cell size. The nuclear DNA contents (2C) in diploid (1.16–1.27 pg), triploid (1.61–2.23 pg) and tetraploid (1.94–1.2.37 pg) *Musa* had large, medium and small genome sizes, respectively (Kamaté *et al*. 2001). Thus, ploidy levels were probably related to pollen size among *Musa* genotypes.

Similar results for viable pollen were also observed for higher pollen tube germination under culture medium condition. Among the studied *Musa* genotypes, both diploid *M. balbisiana* cultivars TKM and TA15 (BB genome composition) and *M. acuminata* cultivars KKP and NT (AA genome composition) had high pollen viability and pollen germination percentage, with the longest pollen tube compared to the other genotypes. This finding was supported by observations that *Musa* AA and BB genomes had high pollen viability and pollen tube germination, with a few differences among them (Damaiyani and Hapsari 2018; Panda *et al*. 2019). This preliminary result suggested that diploid *Musa* cultivars (carrying AA or BB genome) might be useful as male parents in *Musa* breeding programs because both diploids had high percentage of pollen viability and pollen tube germination. These diploid cultivars should also avoid plantations nearby the commercial field of edible *Musa* production (mostly triploid AAA and AAB genomes) to reduce cross-pollination and set seed in their fruit. The role of pollen development-related genes requires further study to better understand the mechanisms of male fertility and sterility in *Musa*.

Molecular aspects of pollen development in flowering plants involve several transcription factors that are tightly regulated by dynamic changes in gene expression (Pearce *et al*. 2015). Up-regulated and down-regulated gene expression profiles are impacted by either fertility or sterility of pollen production (Hu *et al*. 2020). Previous results showed that pollen viability percentages among six studied *Musa* cultivars could be classified into three groups of high (*Musa* T167 and TA15, carrying BB genome), medium (*Musa* KKP and KPB, carrying AA and AAA genomes, respectively) and low (*Musa* NMO and NNJ, carrying ABB genome). The expression profiles of pollen development-related genes were further evaluated using the RT–qPCR assay. *TPD1A*, *MYB80* and *PTC1* were characterized as pollen-specific genes involved in early (microspore mother cell), middle (microspore) and late (mature pollen) pollen development stage, respectively (Parish and Li 2010; Huang *et al*. 2011; Wu *et al*. 2022). Results showed that both the *TPD1A* and *MYB80* genes were highly expressed in the *Musa* triploid genome (ABB and AAA) compared to the diploid genome (AA and BB). By contrast, the *PTC1* gene in the *Musa* triploid genome was less expressed than in the diploid genome. One possible explanation of these gene associations is that up-regulated *TPD1A* and *MYB80* genes in the *Musa* triploid genome might reduce *PTC1* gene expression, resulting in non-viable pollen or abnormal pollen development.

Previous reports suggested that *TPD1A* overexpression in *M. itinerans* (AA genome) promoted *MYB80* gene expression but suppressed *PTC1* gene expression, resulting in absence of pollen in male flowers with the fruit small and seedless (Hu *et al*. 2020). Mutant rice with 25 nucleotide insertions within the *PTC1* gene promoter region showed significantly decreased expression compared to wild-type plants, causing complete pollen sterility (Peng *et al*. 2020). This result suggested that non-viable pollen development in *Musa* might be associated with the up-regulated expression of both *TPD1A* and *MYB80* genes but down-regulated expression of the *PTC1* gene.

## Conclusions

This study assessed 23 *Musa* cultivars for pollen viability using LAO and TTC staining methods. The LAO-stained pollen gave higher viability percentages than TTC-stained pollen. Percentages of pollen viability across all *Musa* cultivars were significantly different. The *Musa* BB genome gave higher percentage pollen viability than the AA, AAA, BBB, AAB, ABB and ABBB genomes, while the *Musa* TKM (BB genome) gave the highest germination percentage assessed by a pollen culture method. For the expression of pollen development-related genes, the *TPD1A* and *MYB80* genes were up-regulated but the *PTC1* gene was down-regulated in the *Musa* triploid genome, resulting in non-viable pollen. Knowledge of pollen viability is important when selecting male breeders for *Musa* cross-breeding programs to prevent seed formation in the fruit.

## Supplementary Material

plad052_suppl_Supplementary_Data

## Data Availability

The raw data have been provided in the Supporting Information, and additional information is available upon request from the corresponding author.

## Notes

**Phenome, Genome and Environment. Chief Editor:** Colleen Doherty

## References

[CIT0001] Adeleke MT , Pillay M, Okoli BE. 2004. Relationships between meiotic irregularities and fertility in diploid and triploid *Musa* L. Cytologia 69:387–393.

[CIT0002] Ahmad F , Poerba YS, Kema GH, de Jong H. 2021. Male meiosis and pollen morphology in diploid Indonesian wild bananas and cultivars. Nucleus 64:181–191.

[CIT0003] Arwatchananukul S , Saengrayap R, Chaiwong S, Aunsri N. 2022. Fast and efficient Cavendish banana grade classification using random forest classifier withsynthetic minority oversampling technique. IAENG International Journal of Computer Science 49:46–54.

[CIT0004] Backiyarani S , Sasikala R, Sharmiladevi S, Uma S. 2021. Decoding the molecular mechanism of parthenocarpy in *Musa* spp. through protein–protein interaction network. Scientific Reports 11:1–14. 34272422 10.1038/s41598-021-93661-3 PMC8285514

[CIT0005] Brewbaker JL , Kwack BH. 1963. The essential role of calcium ion in pollen germination and pollen tube growth. American Journal of Botany 50:859–865.

[CIT0006] Coelho AP , Morais KP, Laughinghouse D IV, Giacomini SJ, Tedesco SB. 2012. Pollen grain viability in accessions of *Crotalaria juncea* L. (Fabaceae). Agrociencia 46:481–487.

[CIT0007] Damaiyani J , Hapsari L. 2018. Pollen viability of 19 Indonesian bananas (*Musa* L.) collection of Purwodadi Botanic Gardens: preliminary study for breeding. In: Proceeding International Conference on Tropical Plant Conservation and Utilization, 42–51.

[CIT0008] De Jesus ON , Amorim EP, Ferreira CF, de Campos JMS, Silva GDG, Figueira A. 2013. Genetic diversity and population structure of *Musa* accessions in *ex situ* conservation. BMC Plant Biology 13:1–22. 23497122 10.1186/1471-2229-13-41 PMC3636076

[CIT0009] De Langhe E , Hřibová E, Carpentier S, Doležel J, Swennen R. 2010. Did backcrossing contribute to the origin of hybrid edible bananas? Annals of Botany 106:849–857. 20858591 10.1093/aob/mcq187 PMC2990659

[CIT0010] Fortescue JA , Turner DW. 2004. Pollen fertility in *Musa*: viability in cultivars grown in Southern Australia. Australian Journal of Agricultural Research 55:1085–1091.

[CIT0011] Ghosh S , Mondal T, Roy A. 2020. Pollen viability study of *Solanum erianthum* D. Don. Journal of Plant Science Research 36:261–265.

[CIT0012] Hu C , Sheng O, Dong T, Yang Q, Dou T, Li C, He W, Gao H, Yi G, Deng G, et al. 2020. Overexpression of *MaTPD1A* impairs fruit and pollen development by modulating some regulators in *Musa itinerans*. BMC Plant Biology 20:1–12. 32867686 10.1186/s12870-020-02623-w PMC7461258

[CIT0013] Huang MD , Hsing YIC, Huang AH. 2011. Transcriptomes of the anther sporophyte: availability and uses. Plant and Cell Physiology 52:1459–1466. 21743085 10.1093/pcp/pcr088 PMC3172567

[CIT0014] Jeensae R , Suvittawat K, Boonruangrod R. 2020. Study on floral structure and pollen morphology of eight Musa genotypes: wild species and cultivars. Songklanakarin Journal of Plant Science 7:184–195.

[CIT0015] Jeridi M , Bakry F, Escoute J, Fondi E, Carreel F, Ferchichi A, D’Hont A, Rodier-Goud M. 2011. Homoeologous chromosome pairing between the A and B genomes of *Musa* spp. revealed by genomic *in situ* hybridization. Annals of Botany 108:975–981. 21835815 10.1093/aob/mcr207 PMC3177683

[CIT0016] Kaewkhumpai A. 2019. *Morphology and effect of temperature on storage life of Npenthes* spp. MS thesis, Prince of Songkla University, Thailand.

[CIT0017] Kamaté K , Brown S, Durand P, Bureau JM, Nay DD, Trinh TH. 2001. Nuclear DNA content and base composition in 28 taxa of *Musa*. *Genome* 44:622–627. 11550896

[CIT0018] Khairul-Anuar MA , Mazumdar P, Lau SE, Tan TT, Harikrishna JA. 2019. High-quality RNA isolation from pigment-rich *Dendrobium* flowers. 3 Biotech 9:1–9. 10.1007/s13205-019-1898-y PMC6761217 31588395

[CIT0019] Martanti D , Poerba YS, Witjaksono H, Ahmad F. 2022. Male flower characteristics of induced tetraploid, mixoploid, and diploid banana *Musa acuminata* (AA) cv. ‘Pisang Rejang’. SABRAO Journal of Breeding and Genetics 54:617–626.

[CIT0020] Munhoz M , Luz CFPD, Meissner Filho PE, Barth OM, Reinert F. 2008. Viabilidade polínica de *Carica papaya* L.: uma comparação metodológica. Brazilian Journal of Botany 31:209–214.

[CIT0021] Ngatat S , Hanna R, Lienou J, Ghogomu RT, Nguidang SPK, Enoh AC, Ndemba B, Korie S, Kuate AF, Nanga SN, et al. 2022. *Musa* germplasm A and B genomic composition differentially affects their susceptibility to banana bunchy top virus and its aphid vector, *Pentalonia nigronervosa*. Plants 11:1206. 35567207 10.3390/plants11091206 PMC9100355

[CIT0022] Nyine M , Pillay M. 2007. Banana nectar as a medium for testing pollen viability and germination in *Musa*. African Journal of Biotechnology 6:1175–1180.

[CIT0023] Ortiz R , Swennen R. 2014. From crossbreeding to biotechnology-facilitated improvement of banana and plantain. Biotechnology Advances 32:158–169. 24091289 10.1016/j.biotechadv.2013.09.010

[CIT0024] Oselebe HO , Nnamani CV, Ikeh E. 2014. Pollen diversity, viability and floral structure of some *Musa* genotypes. Nigerian Journal of Biotechnology 27:21–27.

[CIT0025] Panda AK , Soorianathasundaram K, Vijayakumar RM. 2019. Screening for male fertility status in selected banana genotypes. Electronic Journal of Plant Breeding 10:1309–1316.

[CIT0026] Parish RW , Li SF. 2010. Death of a tapetum: a programme of developmental altruism. Plant Science 178:73–89.

[CIT0027] Pearce S , Ferguson A, King J, Wilson ZA. 2015. FlowerNet: a gene expression correlation network for anther and pollen development. Plant Physiology 167:1717–1730. 25667314 10.1104/pp.114.253807 PMC4378160

[CIT0028] Peng Q , Luchang D, Weilan C, Juan H, Shijun F, Bin T, Jun T, Hua Y, Yuping W, Bingtian M, et al. 2020. A fragment substitution in promoter of *MS92/PTC1* causes male sterility in rice. Rice Science 27:396–404.

[CIT0029] Robles P , Quesada V. 2021. Organelle genetics in plants. International Journal of Molecular Sciences 22:2104. 33672640 10.3390/ijms22042104 PMC7924171

[CIT0030] Šimoníková D , Němečková A, Čížková J, Brown A, Swennen R, Doležel J, Hřibová E. 2020. Chromosome painting in cultivated bananas and their wild relatives (*Musa* spp.) reveals differences in chromosome structure. International Journal of Molecular Sciences 21:7915. 33114462 10.3390/ijms21217915 PMC7672600

[CIT0031] Soares TL , Silva SO, Costa MAPC, Santos-Serejo JA, Souza ADS, Lino LSM, Souza EH, Jesus ON. 2008. *In vitro* germination and viability of pollen grains of banana diploids. Crop Breeding and Applied Biotechnology 8:111–118.

[CIT0032] Soares TL , de Souza EH, Sampaio LFS, de Carvalho Costa MAP, e Silva SDO, dos Santos-Sejero JA. 2015. Effect of collection time on the viability of banana pollen grains. African Journal of Biotechnology 14:1207–1214.

[CIT0033] Soares TL , Souza EHD, Costa MAPDC, Silva SDO, Santos-Serejo JAD. 2016. Viability of pollen grains of tetraploid banana. Bragantia 75:145–151.

[CIT0034] Ssebuliba RN , Tenkouano A, Pillay M. 2008. Male fertility and occurrence of 2n gametes in East African Highland bananas (*Musa* spp.). Euphytica 164:53–62.

[CIT0035] Sukkaewmanee P. 2019. Pollen morphology of native banana cultivar (*Musa acuminata* Colla) in Surat Thani Province. The Journals of Gerontology. Series A, Biological Sciences and Medical Sciences 8:49–52.

[CIT0036] Syakhril , Waluyo B, Kuswanto K. 2019. Aceto-orcein staining for counting somatic chromosomes in castor (*Ricinus communis* L.). Bioscience Research 16:2336–2342.

[CIT0037] Waniale A , Swennen R, Mukasa SB, Tugume AK, Kubiriba J, Tushemereirwe WK, Amah D, Tumuhimbise R. 2021. Application of pollen germination media on stigmas during pollination increases seed set in east African highland cooking bananas (*Musa* spp.). Agronomy 11:1085.

[CIT0038] Wu C , Yang Y, Su D, Yu C, Xian Z, Pan Z, Guan H, Hu G, Chen D, Li Z, et al. 2022. The *SlHB8* acts as a negative regulator in tapetum development and pollen wall formation in Tomato. Horticulture Research 9:1–13. 10.1093/hr/uhac185 PMC9627519 36338846

